# Assessment of Pulmonary Gas Transport in Rabbits Using Hyperpolarized Xenon-129 Magnetic Resonance Imaging

**DOI:** 10.1038/s41598-018-25713-0

**Published:** 2018-05-09

**Authors:** Kai Ruppert, Hooman Hamedani, Faraz Amzajerdian, Yi Xin, Ian F. Duncan, Harrilla Profka, Sarmad Siddiqui, Mehrdad Pourfathi, Stephen Kadlecek, Rahim R. Rizi

**Affiliations:** 1 Department of Radiology, University of Pennsylvania, Philadelphia, PA 19104 USA; 2 Department of Bioengineering, University of Pennsylvania, Philadelphia, PA 19104 USA; 3 Department of Electrical and Systems Engineering, University of Pennsylvania, Philadelphia, PA 19104 USA

## Abstract

Many forms of lung disease manifest themselves as pathological changes in the transport of gas to the circulatory system, yet the difficulty of imaging this process remains a central obstacle to the comprehensive diagnosis of lung disorders. Using hyperpolarized xenon-129 as a surrogate marker for oxygen, we derived the temporal dynamics of gas transport from the ratio of two lung images obtained with different timing parameters. Additionally, by monitoring changes in the total hyperpolarized xenon signal intensity in the left side of the heart induced by depletion of xenon signal in the alveolar airspaces of interest, we quantified the contributions of selected lung volumes to the total pulmonary gas transport. In a rabbit model, we found that it takes at least 200 ms for xenon gas to enter the lung tissue and travel the distance from the airspaces to the heart. Additionally, our method shows that both lungs contribute fairly equally to the gas transport in healthy rabbits, but that this ratio changes in a rabbit model of acid aspiration. These results suggest that hyperpolarized xenon-129 MRI may improve our ability to measure pulmonary gas transport and detect associated pathological changes.

## Introduction

For a comprehensive evaluation of lung function, it is necessary to measure the temporal dynamics of gas transport from the alveolar airspaces into the heart and, ultimately, the circulatory system. However, none of the imaging techniques currently used to detect lung disorders are able to quantify the process of gas transport across the pulmonary capillaries and beyond; in particular, they are unable to measure the degree to which functional impairment of a specific region of the lung contributes to an overall reduction in gas transport. Tools for the characterization of the entire gas transport chain by a single imaging modality could thus provide novel insights into lung function and its degradation by disease. Our purpose in this study is twofold: first, to use a new imaging method to demonstrate how impaired lung function imposes a bottleneck on the gas transport process; second, to demonstrate how this imaging method can be used to quantify the contribution of distinct, functionally impaired lung regions to the total gas transport.

Over the past 20 years, advances in the technology of hyperpolarized noble gas MRI have increased the available magnetic resonance signal approximately 100,000 fold over its thermal equilibrium value, leading to the development of several new diagnostic tools for evaluating regional lung function. Using specifically-designed MRI pulse sequences, images of inhaled hyperpolarized helium-3^1–6^ and hyperpolarized xenon-129 (HXe)^7–14^ have provided many new insights into pulmonary function and structure. Additionally, xenon dissolved in tissue and blood — collectively known as xenon dissolved-phase (DP) — can serve as a surrogate marker for the flow of oxygen throughout the breathing lung and into the circulatory system. Because the associated ~200 ppm frequency shift^15^ allows separate interrogation of gas phase (GP) and dissolved phase dynamics, pulmonary gas exchange may be quantified at the millisecond timescales relevant to healthy organ function. This yields fundamental information about lung function that cannot be accessed in any other way.

Several HXe MRI techniques have been developed for characterizing lung function through the observation of parenchymal xenon gas uptake^16–27^. These HXe DP methods tend to provide a single snapshot of the steady-state distribution of the xenon signal within the lung parenchyma. Because the observed distribution depends on lung physiology, gas transport dynamics and the selected MRI acquisition parameters, regional indications of abnormal or pathological gas uptake may be obtained by choosing an appropriate imaging sequence. Some of these dynamic aspects can also be observed using whole-lung spectroscopic techniques such as chemical shift saturation recovery (CSSR)^28–32^. More recently, efforts have also been made to add coarse spatial information to the CSSR method in order to perform regional measurements of lung function^24^; however, these developments are still in their infancy.

Despite the array of magnetic resonance tools now available for assessing lung function, the problem of linking apparent regional abnormality to a specific reduction in overall lung function persists. Current DP MR imaging methods infer an implicit structure-function relationship in as far as changes in static gas-uptake quantities are believed to be indicative of abnormal functionality. However, although it is reasonable to assume that a complete lack of gas exchange in a parenchymal region has a negative impact on overall function, some heterogeneity and functional compensation among lung regions is expected even in a healthy organ. As a result, abnormality in a static DP map may only indicate different temporal dynamics, with minimal functional impact over the full respiratory cycle.

In this study, we demonstrate how the scope of static DP MR imaging pulse sequences can be expanded to incorporate additional temporal information through modification of the acquisition parameters, providing explicit, model-independent information of lung function. Our methods allow the assessment of whether actual gas transport is taking place—and at what rate—by mapping changes in the DP signal on a pixel-by-pixel basis over the course of a fixed time interval, or by monitoring the response of the DP signal in specific downstream locations to regional destruction of the GP signal. In other words, by using xenon as a surrogate for oxygen, we implemented a much more holistic approach for addressing the complex problem of evaluating pulmonary gas exchange and transport than is currently available through existing techniques. This work therefore provides a strong foundation for the characterization of the gas transport chain through the lung and its perturbation by various forms of lung disease.

## Results

**Figure 1:**
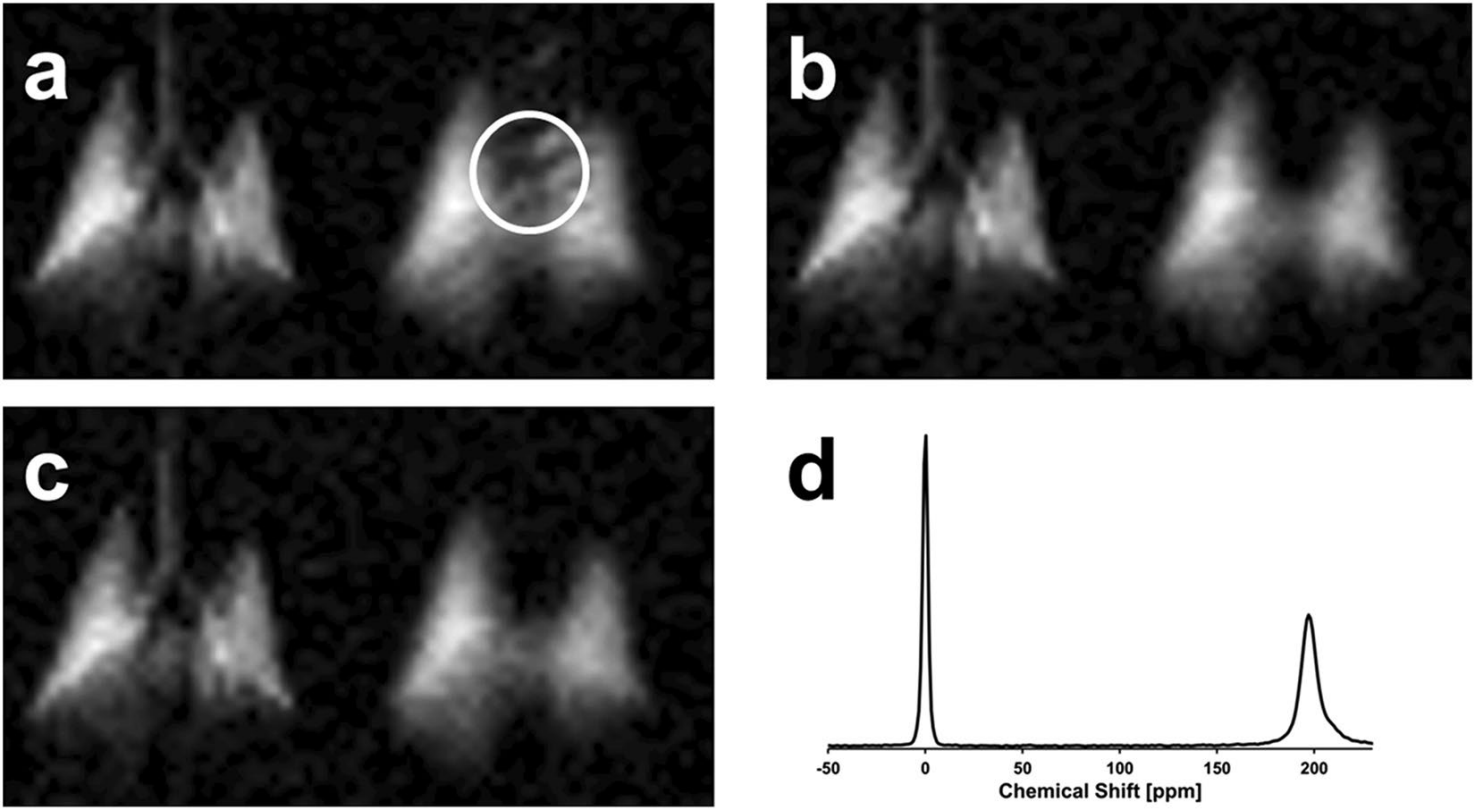
HXe MRI signal distribution in the rabbit lung. (**a**–**c**) Three consecutive, simultaneous GP-DP acquisitions obtained during the same breath-hold using a DP flip angle of 40° for the RF excitation pulse and a TR of 10 ms. The gas image (left side of each panel) and the dissolved xenon image (right side) are shifted apart by their difference in resonance frequency. The DP signal inside the white circle in (**a**) reflects residual downstream signal that had accumulated between the start of the HXe ventilation and the beginning of image acquisition. This signal has been destroyed by the train of imaging RF pulses in subsequent acquisitions. (**d**) Spectrum from the rabbit chest with the GP signal at 0 ppm and the DP peak at 198 ppm. The spectrum is plotted in reverse direction from convention to more naturally correspond to the GP and DP images.

Figure 1 shows a representative study result from a simultaneous GP-DP acquisition (DP flip angle 40°, GP flip angle 1°, 10 ms TR) in a healthy rabbit. All three images (Fig. 1a–c) were acquired consecutively during the same breath-hold. The GP signal is visible on the left-hand side of each panel, while the frequency-shifted DP signal appears on the right-hand side. Despite the 40x greater flip angle at the DP resonance, the GP and DP images have comparable signal intensities due to limited xenon solubility and tissue fraction in the lung. Over the course of a breath-hold, xenon is continuously distributed throughout the body by the blood stream. For DP RF excitation flip angles much lower than 90°, the latter effect is of particular importance at the beginning of the data acquisition, when the distribution of the DP signal is not yet in steady state. As a consequence, some DP signal is visible in the pulmonary veins and the heart in the image collected first (circle in Fig. 1a), but disappears in the subsequent two acquisitions (Fig. 1b,c). Notably, the DP images are not subject to additional chemical-shift blurring due to separate red blood cell and tissue/plasma resonances, as has been observed in humans^19^. As the xenon spectrum of Fig. 1d (acquired in a separate breath-hold from the images of 1a–c) shows, the two DP peaks are unresolved near 200 ppm^33^—similar to mice, but unlike rats, dogs and humans—and are contained within the same pixel for our imaging bandwidth of 110 Hz at 1.5 T.

**Figure 2:**
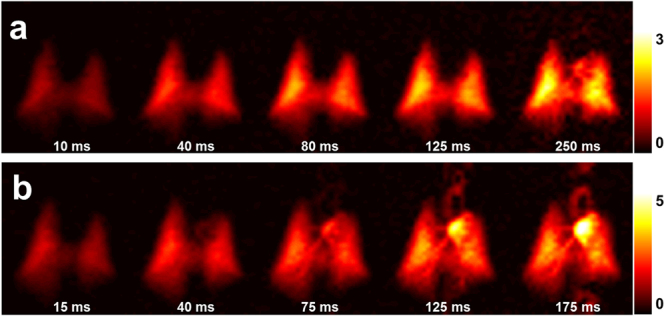
DP xenon accumulation as a function of TR. (**a**) For a DP flip angle of 75°, the DP xenon signal increases throughout the parenchyma as the spacing between RF pulses is lengthened. For a TR of 250 ms, xenon has been transported from the alveolar exchange sites all the way to the left side of the heart. (**b**) A lower DP flip angle of 40° results in reduced depolarization of the DP xenon signal by each RF pulse such that the downstream DP signal accumulates more rapidly. For TRs of 125 ms or more, the aortic arch and possibly the descending aorta become discernible.

Figure 2 illustrates the dynamic aspects of gas uptake in the alveoli and subsequent transport through the vasculature to the heart. For a constant DP flip angle of 75°, the distribution of the DP signal in the chest is strongly dependent on the repetition time (TR) of the image acquisition (Fig. 2a). Using the brightness of the lung parenchyma, major blood vessels, and the left side of the heart as a visual guide, the DP xenon signal is nearly co-localized with the alveolar gas exchange site for short TRs. As the TR is increased, additional time is available for the xenon gas to saturate the lung tissue and follow the circulation to more distal locations. For the first 125 ms of the acquisition with a DP flip angle of 75°, the brightness of the parenchyma gradually increases (indicating saturation of lung tissue and capillary blood); at a TR of 250 ms, a large fraction of the xenon dissolved in the blood has reached the heart, giving rise to a high-intensity signal in the formerly dark cardiac cavity. The measurements depicted in Fig. 2b are equivalent to those in Fig. 2a except for a DP RF excitation flip angle of 40°. The apparent gas transport dynamics for a DP flip angle of 40° are very similar to those for a DP flip angle of 75°, albeit on an accelerated time scale. In Fig. 2b, cardiac DP signal is discernible for a TR of just 75 ms. At the next TR increment of 125 ms, the heart is already brighter than the parenchyma and the aortic arch appears above the heart.

**Figure 3:**
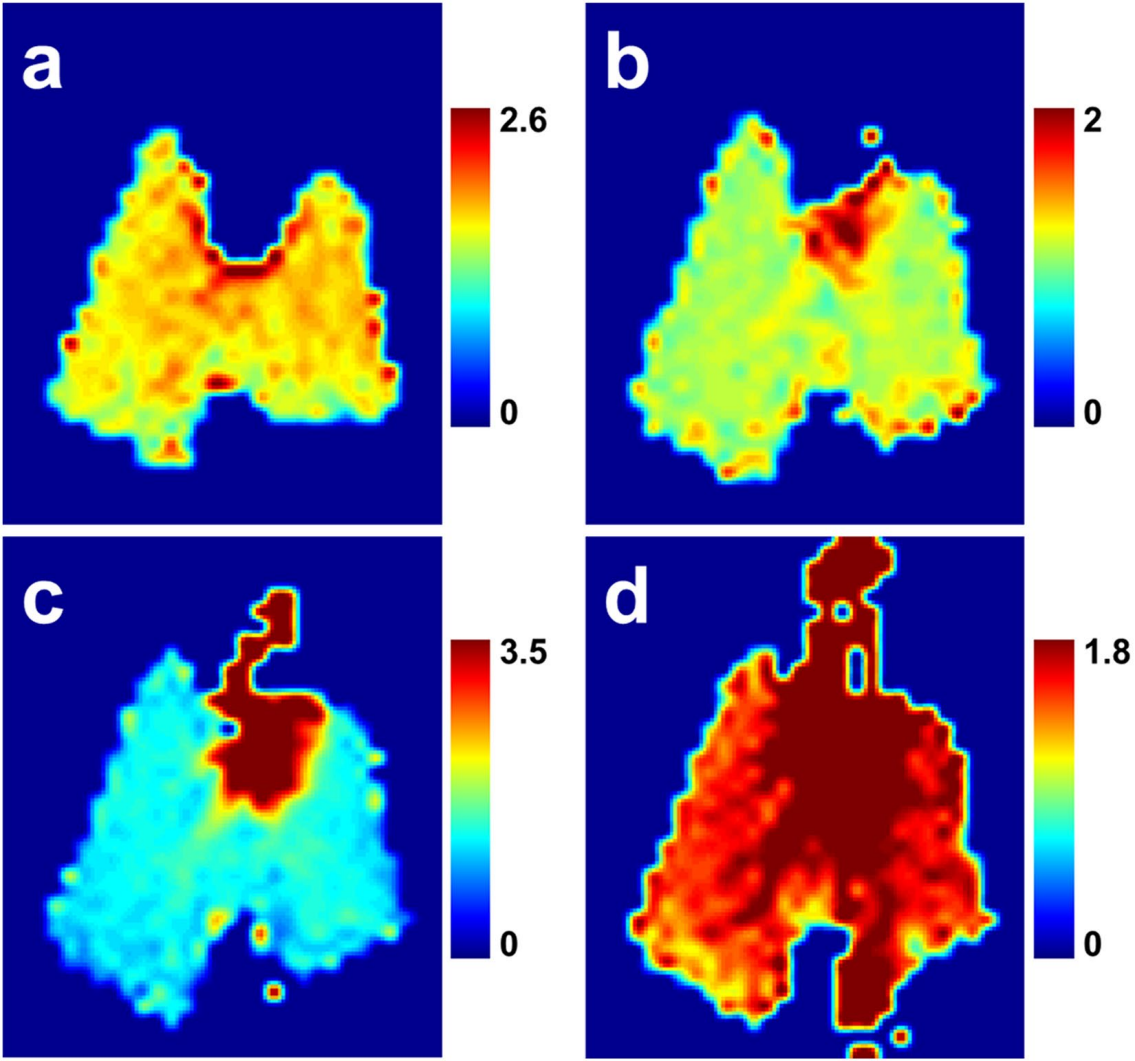
Select DP flip angle and repetition time combinations scaled to illustrate various stages of xenon gas transport during the breathing process. (**a**) Late-stage filling of veins near the heart with xenon-saturated blood (DP flip angle 75°, reference TR 30 ms, comparison TR 125 ms). (**b**) Early arrival phase of DP xenon in the left side of the heart (40°, TR 40 ms vs. TR 50 ms). (**c**) Filling of the aortic arch (40°, TR 40 ms vs. TR 100 ms). (**d**) Full delineation of gas transport through the venous and arterial vessels proximal to the heart (40°, TR 150 ms vs. TR 25 ms).

Juxtaposition of DP maps acquired with different TRs and/or flip angles can be used to highlight various aspects of DP gas transport during the breathing process or at breath-hold. For example, Fig. 3 shows the ratio between maps acquired with a DP flip angle of 75° and TRs of 125 ms and 30 ms: the latter image is used to normalize the former to yield the relative-change map of Fig. 3a. Between these two delay times, the dissolved xenon first reaches the largest pulmonary veins and begins to enter the left heart: the figure therefore highlights late-stage venous filling. In Fig. 3b (DP flip angle 40°, TR 50 ms vs TR 40 ms), the increased DP xenon intensity emphasizes the left atrium and ventricle of the heart, while in Fig. 3c (DP flip angle 40°, TR 100 ms vs TR 40 ms) the xenon-saturated blood has entered the aortic arch. Figure 3d (DP flip angle 40°, TR 150 ms vs TR 25 ms) is scaled to enhance the contrast between the DP in the lung parenchyma and the blood pools in the major vessels and heart. At this setting, second and possibly third order veins become discernible.

**Figure 4:**
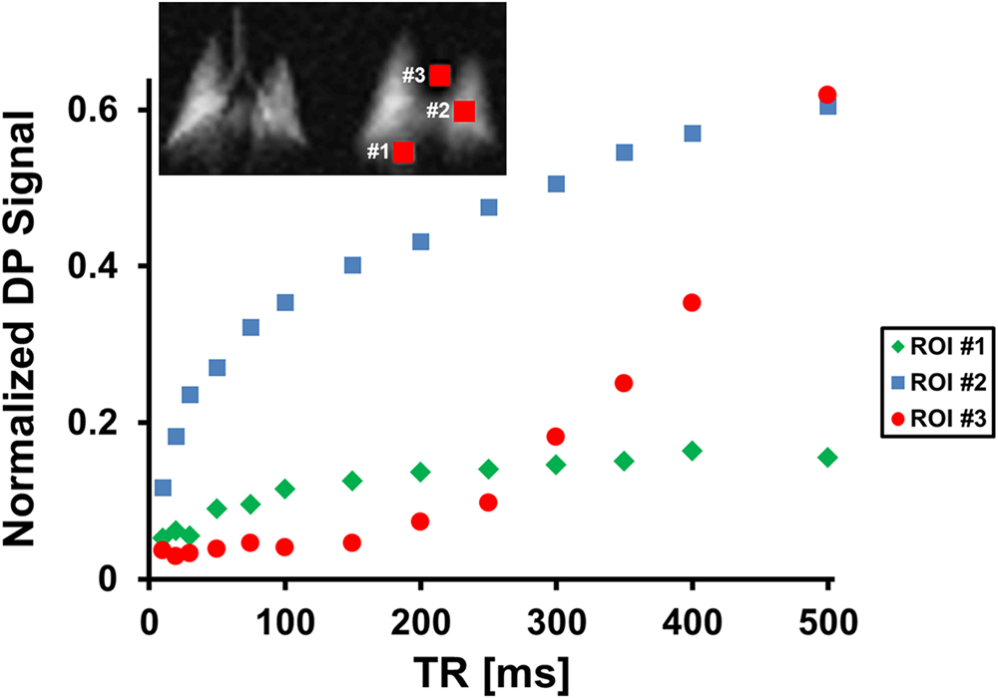
Xenon DP signal as a function of TR in three ROIs and a DP flip angle of 90°. The location of the ROIs relative to the DP lung image is illustrated in the insert. The ROIs exhibit distinct signal characteristics that reflect regional differences in gas uptake and transport.

To allow an initial quantitative assessment of xenon gas uptake and its dependence on TR, we defined 3 regions of interests (ROIs) in the DP images. These were: (1) in the predominantly parenchymal basal posterior section of the right lung, (2) in the centre of the left lung, containing a mixture of parenchyma and large vasculature, and (3) in the left ventricle/atrium of the heart. The location of these ROIs is illustrated in the insert of Fig. 4. The plot in Fig. 4 shows the DP signal behaviour in the selected ROIs as a function of TR with a fixed DP flip-angle of 90°. The signal in ROI #1 increases for approximately 150 ms and then largely levels off, while the signal in ROI #2 continues to rise appreciably. The DP signal from ROI #3 stays at the noise level for about 150–200 ms, at which point the first dissolved xenon gas has travelled from the alveolar gas exchange regions to the heart. After 250 ms, the signal level begins to rise rapidly and approximately linearly with TR.

**Figure 5:**
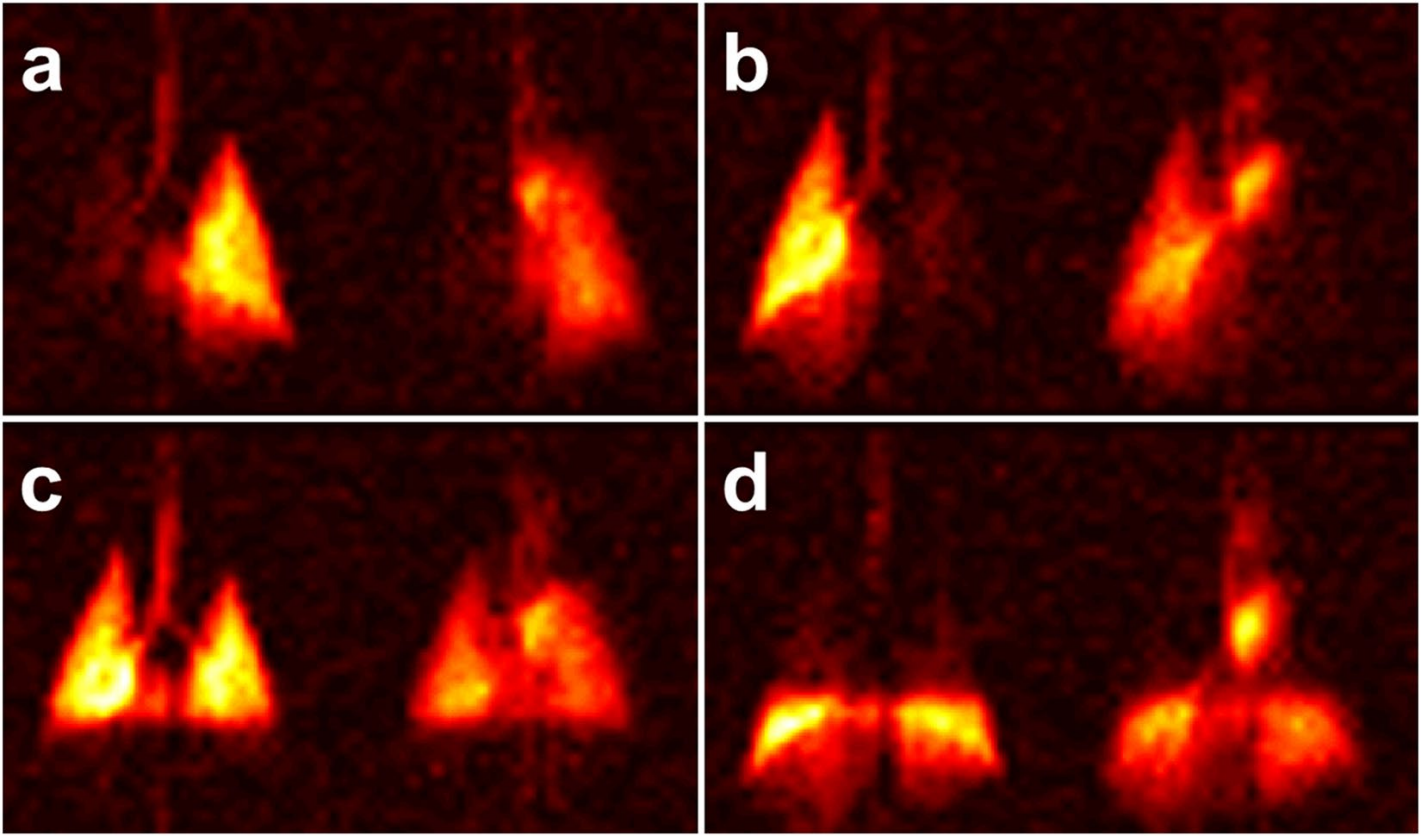
Xenon GP and DP maps for a DP flip angle of 40° and a TR of 200 ms following saturation of the GP signal in the (**a**) right, (**b**) left, (**c**) inferior and (**d**) superior lung. The signal changes in the DP maps relative to an unsaturated baseline map, particularly in a far downstream location such as the heart, reflect the contributions of the saturated lung volume to total lung function.

The contributions from individual parts of the lung to the total pulmonary gas transport can be assessed by saturating the GP signal in the volume of interest (Fig. 5). Here, we used regional RF saturation pulses to destroy the GP signal in the right, left, superior or inferior portions of the lung, which resulted in xenon gas distribution patterns akin to large ventilation defects in the corresponding lung regions. The transition between saturated and unaffected volumes is very sharp and, based on the residual signal in ROIs #1 and #2 (see Fig. 4), approximately 95% of the GP signal is depolarized in the saturation volumes. In the subsequently collected DP images, the xenon signal in the heart (ROI #3 in Fig. 4) dropped by 48% (GP signal change −46%), 42% (ΔGP −41%), 41% (ΔGP −22%), and 32% (ΔGP −40%), respectively. For a GP saturation of the left or right lung arrangements, the DP signal in the heart drops by a similar amount, indicating a good match of ventilation and perfusion in the two lungs of a healthy animal. However, the DP signal decrease in the heart is much higher for inferior saturation and lower for superior saturation than the associated reduction in GP signal would indicate. This is most likely due to the fact that, given the anatomy of the rabbit lung, the saturated inferior lung volume also contains a larger fraction of the total lung parenchyma than the saturated superior lung volume for axial 2D saturation slabs.

**Figure 6:**
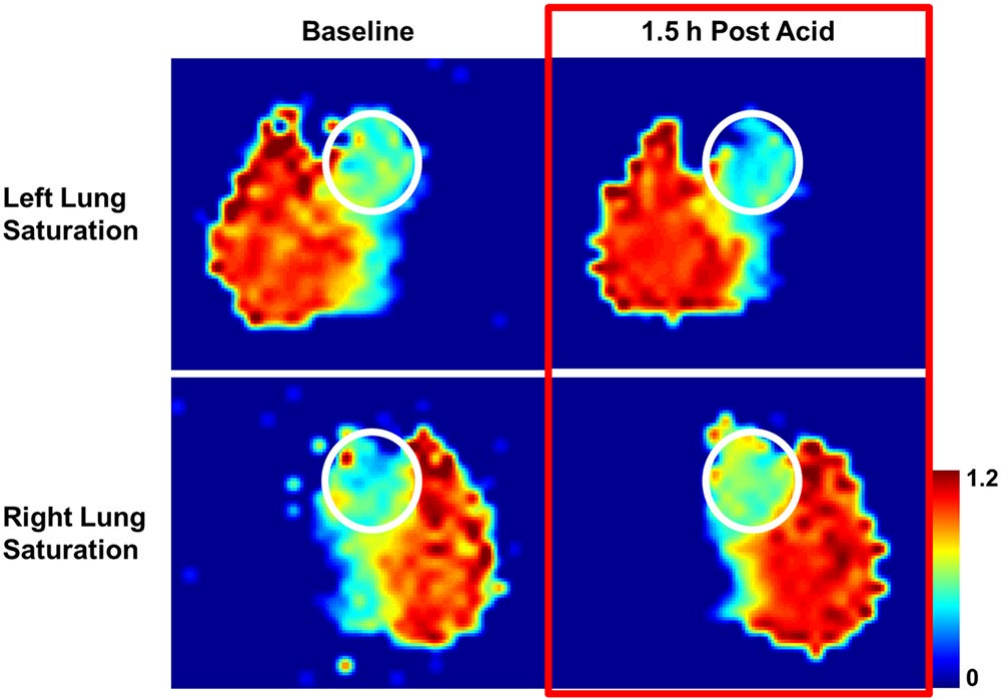
Relative changes in axial projections of the DP signal with and without GP saturation of the left (top row) or right (bottom row) lung at baseline and approximately 1.5 hours after administration of HCl to the right lung. The circles in each map highlight the far-downstream regions, including the heart.

Our hypothesis that injured or diseased portions of the lung have a quantifiably reduced contribution to the total lung function was tested using the one-sided acid aspiration model. After saturation of the right/left lung GP signal, we observed a shift in the relative heart DP signal deficit from slightly right-dominant (52/48%) to significantly left-dominant (46/54%) 1.5 hours after the acid insult (Fig. 6). Similarly, the GP distribution between right and left lung changed from even (50/50%) at baseline to left dominant (46/54%), showing that perfusion followed the ventilation change that is itself likely due to injury-induced alterations of regional lung compliance.

## Discussion

In this study, we demonstrated how the distribution of HXe DP signal in the cardiopulmonary system can be controlled and captured by the choice of DP RF excitation flip angle and the TR of the MRI acquisition. The key feature of our approach is the tight control over the time delay between initial alveolar gas exchange and readout of the HXe DP distribution that the non-renewable nature of the HXe signal permits. In the past, this characteristic has been exploited to measure the rapid xenon gas-exchange processes between the septal walls and the alveolar gas volume. However, using theoretical gas-exchange models^34–37^, the extracted parameters such as septal wall thickness, surface-to-volume ratio, pulmonary transit time, haematocrit, and relative tissue and blood volume were limited to alveolar physiology. Here, we present initial findings which suggest that the downstream gas transport from gas exchange sites through the pulmonary vasculature to the heart and the aorta may provide additional information about lung function that would be difficult to obtain *in vivo* by any other means.

More specifically, the evaluated measurement of the relative changes in DP xenon distribution for different time points subsequent to the actual gas exchange, as controlled by increasing or decreasing the DP excitation flip angle and TR, can potentially identify areas of the lung with degraded gas transport capabilities, providing a powerful tool for the assessment of lung disorders. Relative DP signal changes in the heart following regional destruction of the gas-phase signal allows us to selectively query the contribution of a particular lung volume of interest to the total lung function—something which complex changes in perfusion patterns combined with regional modifications of septal diffusion capacity in the case of disease make it extremely difficult to assess using existing static DP imaging methods. By saturating gas-phase magnetization with an RF pulse, however, such an assessment can be achieved non-invasively without disturbing lung function. In the following, we will discuss the technical details and implications of our conducted studies.

For high DP excitation flip angles approaching 90°, each RF pulse erases almost the entire DP signal, so that the TR of the acquisition determines how much time is available for new xenon gas to be transferred into the lung tissue from the still fully polarized xenon reservoir in the alveolar airspaces. When high excitation flip angles are combined with short TRs, the alveolar xenon can therefore only penetrate the immediate surroundings of the alveoli before the next RF pulse destroys the accumulated DP signal. When the TR is lengthened, the xenon gas initially saturates the parenchyma in the vicinity of the exchange sites, resulting in a fairly homogenous increase in DP signal. At the same time, xenon dissolved in the blood plasma and weakly bound to hemoglobin is carried through the pulmonary capillary bed. Since only about 20% of the lung volume consists of tissue, the DP signal near the gas exchange regions is fairly weak. As the DP xenon travels down the vascular tree and converges in ever larger vessels, however, its signal density increases towards the central regions of the lung (see Fig. 2): for long TRs, the left atrium and the left ventricle of the heart eventually become visible in the images as a new signal source originating between the left and right lung.

The use of high DP excitation flip angles enables the most straightforward assessment of the DP xenon distribution’s time-dependence. However, to fully capture the xenon accumulation in the heart would require TRs of several hundred milliseconds, leading to impractical breath-hold durations and resulting in significant signal loss due to T1 relaxation. To overcome this challenge, it is possible to lower the DP excitation flip angle, dramatically extending the observable temporal range of DP dynamics. Balancing reductions in TR with decreases in flip angle therefore makes it possible to assess far downstream DP signal during reasonable breath-holds shorter than 10 s, as we demonstrated in a set of studies using a DP flip angle of 40° for comparison. In these studies, each RF pulse only destroys about 25% of the DP signal (as opposed to ~75% in the experiments that employed 75° DP flip angles), allowing it to accumulate in the lung tissue for several TRs and causing the TR itself to lose its direct link with the time constants of the gas transport processes. For instance, we found that the DP signal in the heart becomes discernible for a TR around 40 ms with a DP flip angle of 40°, despite the fact that our 90° DP-flip angle studies revealed that it actually takes upwards of 200 ms for the xenon in the blood to travel from the closest exchange sites to the left atrium and ventricle.

The dynamic DP behaviour can be visualized more clearly using relative-change maps. These maps allow us to assess the differences in xenon distribution for different acquisition parameters, and are thus powerful tools for characterizing the changes between various stages of DP xenon transport in the lung that would be very difficult to observe from the individual DP image intensities themselves. Figure 3 depicts a selection of such relative-change maps, each isolating a particular temporal phase of the xenon accumulation in the lung tissue and blood stream from earliest to latest. These measurements differ from what is obtained with existing MRI and CT measurements using contrast agents that mainly measure arterial flow, which cannot be used to generate gas transport maps since they do not measure the rate at which xenon enters the blood stream. Because inhaled hyperpolarized xenon is uniquely capable of tracking the entire gas transport chain of oxygen simultaneously rather than just individual aspects of it, xenon gas transport measurements should therefore map oxygen transport much more closely than either dissolved-phase ratio maps or traditional MR or CT blood pool agents.

Our findings of differences in regional temporal DP dynamics (Fig. 4) are likely to impact the development of spatially-resolved xenon gas-uptake models. Various methods for the quantification of xenon gas exchange and uptake into the blood have been documented in the literature, allowing for the extraction of physiological lung parameters such as alveolar septal wall thickness, capillary transit time, etc., from spectroscopic measurements. Yet the translation of these models to spatially resolved DP signal dynamics^24^ requires further refinement, as a comparison of the three different ROIs in Fig. 4 clearly shows. Unsurprisingly, the xenon gas-transport dynamics are spatially heterogeneous: the DP signal in the distal posterior regions rises quickly, but then appears to reach a saturation point without significant further accumulation; on the other hand, more centrally located volumes exhibit temporal behaviours that, at least qualitatively, closely mimic the two-compartment dynamics for which the existing models have been designed. These curves consist of an initial gas exchange phase during which xenon saturates the alveolar septal walls and the capillary blood pool, followed by a linear transport phase as the dissolved xenon is carried downstream by the pulmonary circulation. As one would expect, the lack of such a distinct transport phase indicates the absence of larger downstream veins in ROI #1 (see Fig. 4), while the strong presence of a transport component in ROI #2 leads to the opposite conclusion. Finally, the heart displays yet another signal behaviour: almost no DP signal is detectable for the first 150 ms, until the dissolved xenon begins to fill the major veins surrounding the heart and then the left side of the heart itself. Global gas-uptake models that do not take into account this highly heterogeneous regional vascularization are therefore unsuitable for dynamic DP image acquisitions.

The only mechanism by which DP signal can appear in the cardiac cavity is through uptake in the alveoli and transport by the blood stream. This means that the xenon DP signal in the heart is reflective of the amount of gas exchange that has taken place in the entire ventilated lung volume over the course of a certain period of time as determined by the flip angle and the TR of the image acquisition. This circumstance permits a completely unexplored approach for the characterization of xenon gas transport by using the DP signal in the heart as a reference point. If the GP signal is destroyed by a regional GP RF saturation pulse prior to the start of the pulse sequence, the DP signal in the heart will drop proportionally to the contribution of the saturated lung volume to the total gas transport. Saturating the GP in a certain lung volume and observing the impact on the heart signal therefore permits the quantification of lung function in that particular volume. In a healthy rabbit, we found that the left and right lungs contribute almost equally to the gas uptake by the lung as a whole. However, this is no longer the case in heterogeneous lung disease or local lung injury.

One potential drawback of using rabbits in our experiments is the fact that, unlike in humans, the DP resonance frequencies for xenon dissolved in lung tissue/blood plasma and bound to haemoglobin in red blood cells overlap enough that they cannot be resolved in separate distribution maps. However, this separation is immaterial for measuring the DP signal behaviour in the heart. Since the focus of this work was to demonstrate for the first time the temporal dynamics of pulmonary gas uptake and transport, rabbits in fact offer an ideal model, since both compartments have both an uptake and a transport component, and must therefore be analysed together.

In this work, we have demonstrated how the acquisition parameters of a suitably-designed HXe MRI pulse sequence can be harnessed for tracing, and potentially quantifying, pulmonary gas transport processes. We anticipate that the specific MRI parameters for capturing particular transport stages would be species-dependent, but should follow the observed trends regardless of subject size. Since the DP images that we used to calculate relative-change maps are currently acquired during separate breath-holds, their comparison may be subject to minor registration errors. In the future, however, two or possibly more parameter permutations could be acquired during the same breath-hold in an interleaved, or at least consecutive, fashion. Techniques based on evaluation of the DP signal in the heart or other organs following regional GP saturation may prove to be of particular interest for assessing the relative contributions of these regions to total lung function or tissue oxygenation—e.g., following lung transplantation or pharmaceutical intervention—as well as for surgical planning. However, until disease-specific research addresses the applicability and sensitivity of this technique, their ultimate clinical or research utility remains unknown. Nonetheless, it is not too much of a stretch to imagine how it might be useful clinically. While determining the regional contribution to blood oxygenation is one of the most important aspects of our method, it leaves some ambiguity about the cause of any observed abnormalities. Additional knowledge about uptake and vascular transport times, or their heterogeneity inside the interrogated region, may help determine this cause— or at the very least indicate further specific tests of function or structure that are likely to provide greater clarity. On the other hand, a lung characterized by homogeneous function and normal transport dynamics might quickly be eliminated as the potential cause of patient morbidity.

## Methods

### Animal Studies

Experiments were performed using five New Zealand rabbits (approx. 3.1–4.5 kg each). The rabbits were sedated with 25–35 mg/kg ketamine and 5 mg/kg xylazine IM, and 1–5% isoflurane was administered through a mask to maintain deep anaesthesia during animal preparation. A peripheral vein was cannulated to maintain general anaesthesia during imaging. After anesthetization, a tracheotomy tube was inserted using an aseptic surgical procedure and was secured using a silk ligature. 0.75 cc/kg HCl (pH 1) was instilled into the right lung of one animal to mimic gastric acid aspiration. In this model, intended as an initial test of our imaging technique’s sensitivity to lung injury, imaging was performed at baseline and 1.5 hours after acid administration.

After induction of anesthesia, animals were placed in a xenon RF coil (described below) and attached to a home-built, MRI-compatible mechanical ventilator. Anesthesia was maintained by an infusion of Propofol (20–80 mg/kg/h) throughout the imaging session, and body temperature was supported by a circulating warm water pad. Animals were euthanized at the end of the imaging procedures. All experiments were approved by and performed in accordance with the guidelines established by the University of Pennsylvania Institutional Animal Care and Use Committee (IACUC) and the NIH guidelines for the care and use of laboratory animals.

Imaging was performed on a 1.5-Tesla commercial whole-body scanner (Magnetom Avanto; Siemens Medical Solutions, Malvern, PA, USA) that had been modified by the addition of a broadband amplifier, permitting operation at the resonant frequency of 17.6 MHz. The RF coil was a custom xenon-129 transmit/receive birdcage design (Stark Contrast, Erlangen, Germany), positioned to cover the whole chest of the animal. Low-resolution proton MR scout images were obtained with the built-in body coil.

### Gas Polarization and Administration

Enriched xenon gas (87% xenon-129) was polarized by collisional spin exchange with an optically pumped rubidium vapor using a prototype commercial system (XeBox-E10; Xemed, LLC, Durham, NH) that provided gas polarizations of 40–50%. Immediately before MR data acquisition, 1.25 L of HXe gas was dispensed into a Tedlar bag (Jensen Inert Products, Coral Springs, FL) inside a pressurizable cylinder that was subsequently connected to and controlled by the ventilator. Animals were ventilated with room air until the beginning of the imaging study, when the gas mix was switched to 20% oxygen and 80% HXe for two breaths (6 ml/kg tidal volume). After the second inhalation, ventilation was suspended for 5–15 s and image acquisition was started.

### Simultaneous 2D-Projection Gas-Phase/Dissolved-Phase Acquisition

DP xenon resonances are separated from the GP resonance in the lung by approximately 200 ppm. It is therefore feasible to image either one of these two distinct regions individually without signal contamination from the other, either by selective excitation with a low-bandwidth RF pulse or by exciting all resonances simultaneously but sampling with a sufficiently small bandwidth that the DP and GP images do not overlap. To achieve the latter, we implemented a 2D projection acquisition similar to the one described in detail by Mugler *et al*.^19^. Briefly, the pulse sequence is based on a standard RF-spoiled gradient echo sequence with a non-selective 900-μs Gaussian RF excitation pulse centred 200 ppm downfield from the gas resonance that predominantly excites the DP region. However, the RF pulse was sufficiently short to excite the GP resonance as well, albeit with a flip angle 2.5% that at the DP resonance. This scaling relationship was established through a calibration acquisition that measured each k-space line twice: once with a 40° excitation pulse centred at the DP resonance, and once with a 2° excitation flip angle centred at the GP resonance. The GP signal for 2° GP excitation was twice as large as for the 40° DP excitation.

To destroy any DP signal taken up prior to the data collection, the sequence was preceded by two 900-μs Gaussian RF saturation pulses also centred at 200 ppm. The sampling was 65% asymmetric with a bandwidth of 110 Hz, which at the main field strength of 1.5 T and a gyromagnetic ratio for xenon-129 of 11.78 MHz/T yielded a 32-pixel separation between the GP and DP images in the readout direction. Other sequence parameters included: matrix size 36 × 80 (interpolated to 144 × 320); TR/TE 10–500/2.7 ms; FOV 238 mm; flip angle at 200 ppm 40°, 75° or 90°. Up to 3 images were acquired consecutively during each breath-hold using identical imaging parameters. For some of the studies, regional GP saturation was performed using 50 mm slabs that covered approximately half of the lung (left, right, superior or inferior).

### Data analysis

All image reconstruction, post-processing and data analysis was performed using customized MatLab (Mathworks, Natick, MA, USA) scripts. The asymmetrically sampled k-space data was filled using a Homodyne algorithm^38^ before Fourier transform. Each GP-DP image set was normalized by the average signal intensity in the GP image component. The lung was segmented from the background noise by thresholding. The mean signal intensity was computed in three 3 × 3 pixel regions of interest (ROIs), chosen to highlight the xenon buildup behaviour characteristic of different cardiopulmonary structures. The total GP signal in the segmented lung region before and after regional GP saturation was calculated as a reference for the saturation-induced change in the DP signal in the heart (ROI #3). For the calculation of the relative change maps, two thresholded DP image components of interest were extracted, and their amplitudes divided on a pixel-by-pixel basis. The value in a given map was capped to the maximum number displayed next to the scaling bar to achieve the desired dynamic range and contrast.

### Data availability

The datasets generated and/or analysed during the current study are available from the corresponding author on reasonable request.
